# Chronic and Cumulative Adverse Life Events in Women with Primary Ovarian Insufficiency: An Exploratory Qualitative Study

**DOI:** 10.3389/fendo.2022.856044

**Published:** 2022-06-23

**Authors:** Junyan Sun, Yihui Fan, Ying Guo, Huiying Pan, Chen Zhang, Guoping Mao, Yating Huang, Boning Li, Tingting Gu, Lulu Wang, Qiuwan Zhang, Qian Wang, Qian Zhou, Bai Li, Dongmei Lai

**Affiliations:** ^1^ The International Peace Maternity and Child Health Hospital, School of Medicine, Shanghai Jiao Tong University, Shanghai, China; ^2^ Shanghai Key Laboratory of Embryo Original Diseases, Shanghai, China; ^3^ Shanghai Municipal Key Clinical Specialty, Shanghai, China; ^4^ Centre of Exercise, Nutrition and Health Sciences, University of Bristol, Bristol, United Kingdom

**Keywords:** primary ovarian insufficiency (POI), adverse life events, workplace stress, family stress, sleep problems

## Abstract

**Background and Purpose:**

Primary ovarian insufficiency (POI) has serious physical and psychological consequences due to estradiol deprivation, leading to increased morbidity and mortality. However, the causes of most POI cases remain unknown. Psychological stress, usually caused by stressful life events, is known to be negatively associated with ovarian function. It is important to explore high-frequency adverse life events among women with POI for future interventions.

**Methods:**

Forty-three women (mean age=33·8 years) were recruited who were newly- diagnosed with idiopathic POI (FSH levels >40 IU/L) to participate in semi-structured interviews through convenience sampling. The main questions covered by the topic guide were designed to explore adverse life events prior to POI diagnosis. Interviews were audio recorded, transcribed and analyzed thematically. Data were analyzed from June 2019 to August 2020.

**Results:**

Among the women with POI, mean age at diagnosis of POI was 33·8 years (range from 19 to 39 years), and the average time between the onset of irregular menstruation and POI diagnosis was 2.3 years. These women with POI had a relatively normal menstrual cycle before the diagnosis. A number of stressful life events prior to POI diagnosis were discussed by them as important factors influencing their health. Four core themes emerged: 1) persistent exposure to workplace stress, 2) persistent exposure to family-related adverse life events, 3) sleep problem/disturbance existed in women with POI before diagnosis, and 4) participants’ general cognition and concerns about POI.

**Conclusions:**

Persistent exposures to adverse life events related to work stress, family stress and sleep problem existed in women with POI. Our findings are consistent with the hypothesis that adverse life events play a role in the development of POI. Future research should investigate how social environmental factors influence POI disease risks, and whether provision of tailored interventions (i.e. preventing or mitigating impact of adverse life events) aimed at high-risk populations may help prevent new POI cases and improve conditions of women with POI. We gained an in-depth understanding of the experiences of these women *via* 1:1 qualitative method, and find adverse life events are frequent in women with POI prior to the diagnosis.

## Introduction

Primary ovarian insufficiency (POI) is the occurrence of hypoestrogenic amenorrhea in women under the age of 40 years, which is defined as oligo/amenorrhoea for at least four months and menopausal serum FSH levels (> 25 IU/l) on two occasions at least 4 weeks apart before the age of 40 (1, 2). The prevalence of POI in the overall population is approximately 1% (3). POI has various physical and psychological consequences due to early estradiol deprivation, leading to increased morbidity and mortality (4). Compared with women who had menopause at age 50-51 years, women with POI and early menopause had a substantially increased risk of cardiovascular disease (5).

POI is most commonly due to follicle dysfunction inappropriate luteinization of follicles rather than follicle depletion (6, 7). Multiple causes have been recognized, including genetic abnormality, autoimmunity, and iatrogenic mechanisms (3). However, only 10% of POI cases could be explained by known reasons as mentioned above. Among them, genetic etiology has been of great concern. The most common genetic causes of POI are Turner syndrome, premutation in the fragile X messenger ribonucleoprotein 1 (*FMR1*) gene, somatic and X chromosome gene defects (*FOXL2*, *eIF4ENIF1*, *STAG3*, *NR5A1*, *BMP15*, *FSHR*, Gs alpha, and steroidogenic enzymes) (8). In a review of recent advance in POI understanding and management from Yale university, over 60 mutations were validated in several genes (such as *MCM8/9*, *NOBOX*, *GDF9*, *BRCA1/2, FOXL2, FIGLA, BMP15, NANOS3*, and *STAG3*) as causative of idiopathic POI through the rapid development of gene screening technology including array comparative genomic hybridization (CGH), genome-wide association studies (GWASs) and genome-wide sequencing *via* next-generation sequencing (NGS). However, the coding mutations found can explain only a minority of cases, while many other genes have been implicated (9). Thus, exploring unknown potential risk factors in the development of POI is essential.

Psychosocial stress caused by stressful life events, has been associated with increased susceptibility to many diseases, such as cardiovascular disease, neurodegenerative disorders, and cancers (10, 11). Stressful/adverse life events are defined as those that are harmful or threatening to physical health and are likely to be cumulative with each event adding to the total stress burden (12). A prospective cohort study has showed that psychological stress is associated with a longer time-to-pregnancy, an increased risk of infertility and a negative effect on the outcome of *in vitro* fertilization (13). Psychological stress is also negatively correlated with serum anti-Mullerian hormone (AMH) levels, introducing as a marker of ovarian response, in infertile women (14). Higher psychological stress is also associated with an accelerated reduction in antral follicle count (AFC) in pre-menopausal women (15).

As doctors working with women of POI, we frequently encounter questions about the etiology of the disease. These have included for example ‘is my disease related to my overwhelming workplace stress?’ and ‘is it to do with my habit of staying up late?’. However, it is unknown whether stressful/adverse life events are linked to POI. In this study, a qualitative method was first applied to study whether adverse life event is a potential risk factor for idiopathic POI.

## Methods

### Participant Recruitment

We recruited participants using purposive sampling at outpatient clinic in an urban tertiary specialized hospital in Shanghai, China between 1st of August 2018 and 20th of May 2019. The recruitment criteria included newly diagnosed POI (oligo/amenorrhoea for at least four months and FSH in the menopausal range levels >40 IU/L on two occasions > 6 weeks apart before the age of 40) with unknown causation. The exclusion criteria included: 1) abnormal chromosome and/or *FMR1* premutation; 2) iatrogenic factors with a known relationship with POI, such as radiotherapy, chemotherapy and gynecological operation, and 3) autoimmune disorders including thyroid disease, systemic lupus erythematosus, Addison’s disease, or positive of thyroid peroxidase antibody (TPO-Ab) and/or adrenocortical antibody (ACA).

### Research Ethics

This study complies with the Declaration of Helsinki (16). Ethical approval was obtained from International Peace Maternity and Child Health Hospital (IPMCH) Ethic Committee [No (2015).26]. Informed consent (with information on study purpose and the roles of voluntary participants) was sought for all participants prior to the commencement of the interview.

### Data Collection

We used in-depth, semi-structured interviews to explore experience of adverse life events in women newly- diagnosed with idiopathic POI.

We developed a topic guide to facilitate discussions on participants’ experiences in relation to four broad topics: 1) lifestyle and behavioral factors (e.g., smoking, alcohol consumption, diet, and physical activity), 2) chemical agents (e.g., pesticides, industrial pollutants, and medications), 3) radioactive exposure (e.g., radiation from medical and other environmental sources), and 4) social and cultural influences (e.g., family, community, psychosocial/social, and societal factors). We then revised the topic guide after testing it with five volunteers who met the inclusion criteria. This piloting process informed four broad topics: occupational stress, family stress, life style and perceptions of POI (**Supplementary File**:Topic guide).

All the interviews were conducted in a private room by the same researcher (DML) who was fully trained in the qualitative data collection. All interviews were audio recorded and field-notes were taken for each interview to record finer interactions between the participant and the researcher and any relevant contextual information. Each interview lasted for approximately 60 to 90 minutes. At the end of each interview, the interviewer summarized events discussed by the interviewee to check the understanding of the interviewer and whether the interviewee would likely add anything. Collection of qualitative data was continued until no new data emerged to ensure data saturation.

To complement the qualitative data, we utilized the Life Event Scale (LES) that has been validated in a Chinese population to help each subject to recall more reliably over extended periods of time and to define adverse life events (17). Specifically, each interviewee was asked to quantify the degree to which each experience affected their emotion on a 3-level scale (little, moderate, or high impact). Stressful life events were described in the study sample both by ‘event prevalence’ and ‘percentage of cases leading to a perceived moderate/high impact’.

### Data Analysis

All interviews audio data were transcribed verbatim. Qualitative data analysis software (QDA Miner Lite, Provalis Research, Montreal, Quebec, Canada) was used. Transcripts were systematically and thoroughly read and coded by the interviewer with her field notes to inductively identify initial themes which evolved as coding was continued. Coded data was then discussed, compared, and refined to identify core themes. These themes were reviewed to evaluate how well they captured the coded data, and how far they reflected the entire data set. Moreover, a prevalence-impact score of stressful life events in LES was calculated by multiplying event prevalence by the percentage of moderate/high impact (18). This study is presented in line with consolidated criteria for reporting qualitative research (COREQ) guidelines (19). The clear regional boundaries (i.e. the city of Shanghai), the qualitative approach bear the risk of identification of study participants in case of a provision of individual data. Thus, in accordance with the informed consent and the data protection regulations, data can only be made available upon request to the author.

## Results

A total of 43 women with POI took part in this study, the mean age at diagnosis of POI was 33.8 years (range from 19 to 39 years). Mean BMI was 20.7 (range from 16.01 to 29.52), 7% were overweight or obese. 69% attained a college or graduate degree. 72% were married, and 14% were divorced or separated. No one reported a smoking habit but one (2%) was alcoholic. Moreover, 12% of these women with POI reported to engage in physical activities regularly. 93% reported no other medical history, whereas 6% reported a medical history of gallstone disease, acute pyelonephritis, or depression (**Table 1**).

**Table 1:** Baseline characteristics (n=43).

Age (years old)	
Mean	33·8 (SD=5·2)
BMI (kg/m^2^)	
Mean	20·7 (SD=2·7)
Education	
Less than high school	5% (2)
High school	26% (11)
College	60% (26)
Graduate degree	9% (4)
Marital status	
Single	14% (6)
Married	72% (31)
Divorced/separated	14% (6)
Occupation	
Student	2% (1)
Working woman	93% (40)
Housewife	5% (2)
Lifestyle	
Smoking	None
Excessive alcohol drinking	2% (1)
Regular physical exercise	12% (5)
Anxious and nervous personality	54% (23)
Medical history	
Gallstone disease	2% (1)
Acute pyelonephritis	2% (1)
Depression disorder	2% (1)

BMI, body mass index.

As shown in **Table 2**, 79% of the participants had menarche at age 12-15 years old and had a relatively normal menstrual cycle before the diagnosis of POI, and the average time from irregular menstruation to POI diagnosis was 2.3 years. 26% of the participants experienced one-time induced abortion and 22% had two or more abortions. Spontaneous abortion occurred to 12% of these women with POI. 35% of the women had not given birth to a child at the time of POI diagnosis. In addition, the mean menopausal age of their mother was 50 years old. Serum hormone concentrations of these participants were analyzed as follows: FSH= 87·34 ± 33·85mIU/ml (95% CI: 77·22 to 97.46mIU/ml); LH=41·91 ± 18·71mIU/ml (95% CI: 36·44 to 47·37mIU/ml), E2 = 154·51 ± 127·91pmol/L (95% CI: 110·19 to 198·83pmol/L, 23% of the participants with E2 value < 20pmol/L), and AMH=0·26 ± 0·5 ng/ml (95% CI: -0·07 to 0·59ng/ml, 35% of the participants with AMH value < 0·06 ng/ml).

**Table 2:** Reproductive characteristics and endocrine profiles (n = 43).

Age at menarche (years old)	
≤11	9% (4)
12—15	79% (34)
≥15	11% (5)
History of menstrual cycle (days)	
<25	12% (5)
25-35	86% (37)
>35	2% (1)
Abortion	
One-time induced abortion	26% (11)
Two or more times induced abortion	22% (9)
One-time spontaneous abortion	12% (5)
Parity distribution	
No child	35% (15)
One child	51% (22)
Two or more children	14% (6)
Level of hormone	
FSH (mIU/ml)	87·34 (95%CI: 77·23-97·46)
LH (mIU/ml)	41·91 (95%CI: 36·44-47·37)
E2 (pmol/l)	
Mean	154·51 (95%CI: 110·19-198·83)
<20	23% (10)
AMH (ng/ml)	
Mean	0·26 (95%CI: -0·07-0·59)
<0.06	35% (15)
Time from irregular menstruation to diagnosis of POI (years)	
Mean	2·3 (SD=2·05)
Menopausal age of mother (years old)	
Mean	50 (SD=3)
No menses with hysterectomy history	14% (6)
Unknown	12% (5)

FSH, follicle-stimulating hormone; LH, luteinizing hormone; E2, estradiol; AMH, anti-müllerian hormone.

**Figure 1:**
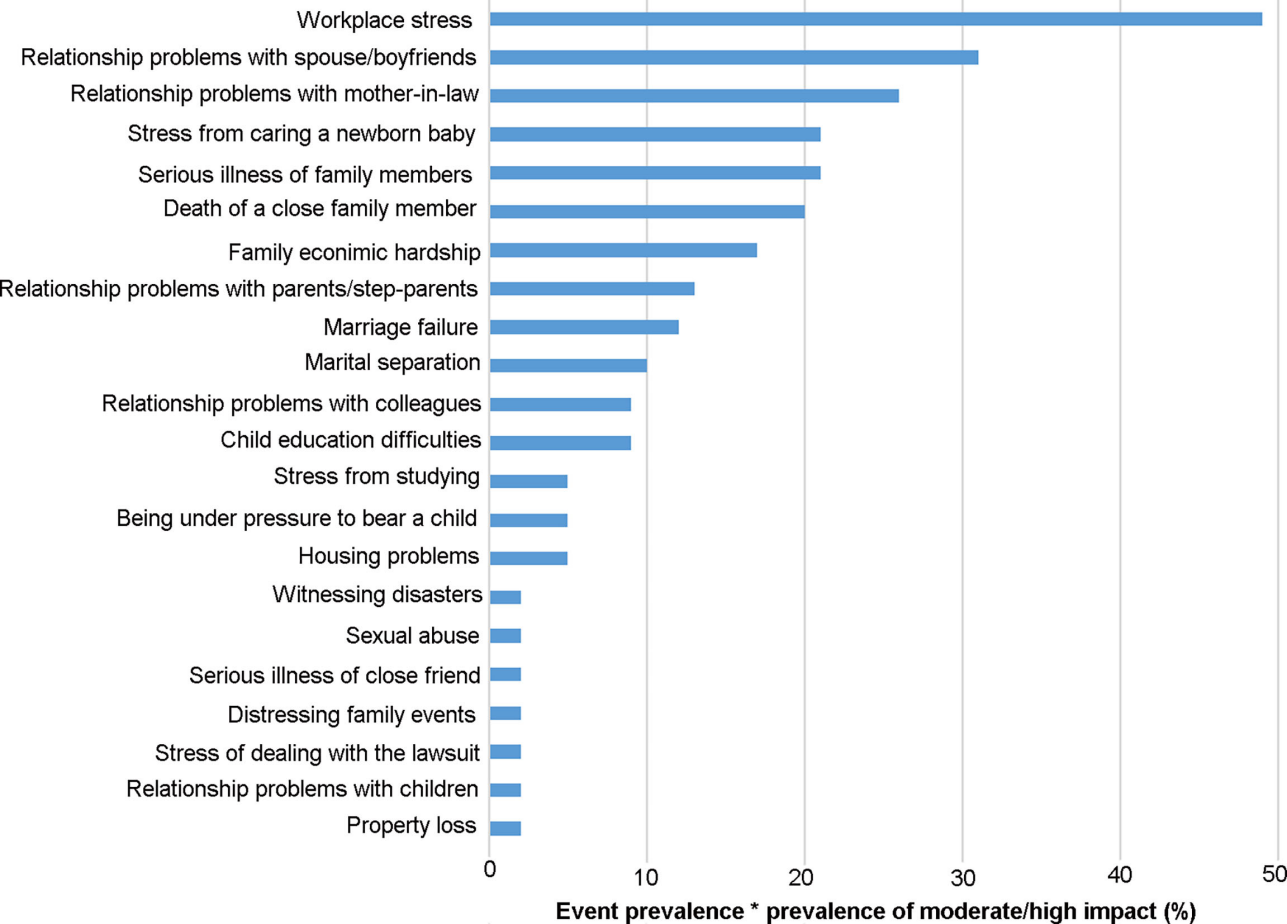
Stressful life events with the prevalence-impact scores. The prevalence of stressful life events and the frequency of moderate/high impact is treated as percentages, and their product is multiplied by 100 to obtain the frequency-impact score.

Adverse life events prior to POI diagnosis were discussed intensively by all interview participants. Twenty-four adverse life events were self-reported by participants with adverse effect on themselves, the top ten adverse life events self-reported by participants with moderate/high adverse effect on themselves were “workplace stress”, “relationship problems with spouse/boyfriend”, “relationship problems with mother-in-law”, “stress from caring a newborn baby “, “serious illness of family members”, “death of close family members”, “family economic hardship”, “relationship problems with parents/step-parents” “marriage failure” and “marital separation”(**Figure 1**). Some adverse life events were reported less frequently, such as serious illness of a close friend, sexual assault, property loss and witnessing disasters. In addition, 7% of the women reported significant weight loss with dieting, but they thought it had little effect on themselves (**Figure 1**). Four core themes emerged. These were reported below with illustrative quotes.

### Core Theme 1: Persistent Exposure to Workplace Stress

Most participants recalled persistent, adverse experience of workplace stress and believed this to be a major cause of their diagnosed POI condition. In addition, experiences related ‘night shift’ and ‘relationship problems with colleagues’ were widely discussed. Consistently, our complementary questionnaire identified 24 types of stressful life events among the interview participants (**Figure 1**). Of these, ‘workplace stress’ was the most frequently reported type of adverse life events. The following quote was from a participant who experienced menstrual disorder at the age of 30 and was diagnosed POI at 31 years old.

“When I was 27 years old, I set up my own company and managed everything by myself. And I had been in a state of high mental tension for three years because the company was in its early developmental stage. At that time, I always went to bed late, even could not fall asleep overnight. I thought I had taken on too many responsibilities and I shouldn’t have had that much of pressure at that age.” (Participant 1)

This woman experienced menstrual disorder at the age of 30 and was diagnosed POI at 31 years old.

### Core Theme 2: Persistent Exposure to Family- Related Adverse Life Events

Psychological and physical stress derived from their families were mentioned by most participants, including relationship problems, serious illness or death of family members, marital separation, marriage failure, stress from caring a newborn baby (especially for chronically ill children), child education difficulties, family economic hardship and housing problems. Many participants believed that family related adverse life events might have had a bigger impact on their health than workplace related stressful events did. Some of the participants could not help weeping during the interview. Familial relationship problems included relationship with spouse/boyfriend, parents/step parent, mother-in law and children. As stated by one participant (*Participant 29*, menstrual problems occurred at 38 years old and was diagnosed with POI at 39),

“I had relationship problems with my husband for more than five years. The “cold war” continued and there was no intimacy between us. My mother-in-law was interfering too much with our life when she lived with us, my husband thought she was doing great: she cooked dinner and took care of our kid. I was not satisfied with her at all. We could not agree with each other, so the cold war kept going. This family conflict has impacted me significantly. I even did not want to go home after work, and I was in a depressive mood. My friends urged me to see a doctor. But, I did not, because I doubted they could help me.”

Some adverse life events, such as housing problem, was less frequently discussed but was believed to be a major cause of POI in some participants. As one woman shared (*Participant 17*, developed menstrual disorder at 35 and was diagnosed with POI at 37),

“Eight people lived together in my family in a two-bedroom apartment of 60 square meters for eight years. It was already quite crowded. Then my cousin moved to my house since she did not like the dormitory. There were only three fixed beds, and we had to assemble the other beds at night and dissembled them in the morning. Nine of us had lived that way for about one year. Considering the peace of family, I have endured them all the time, however, I always felt constrained over the past nine years.”

### Core Theme 3: Sleep Problem/Sleep Disturbance

**Figure 2:**
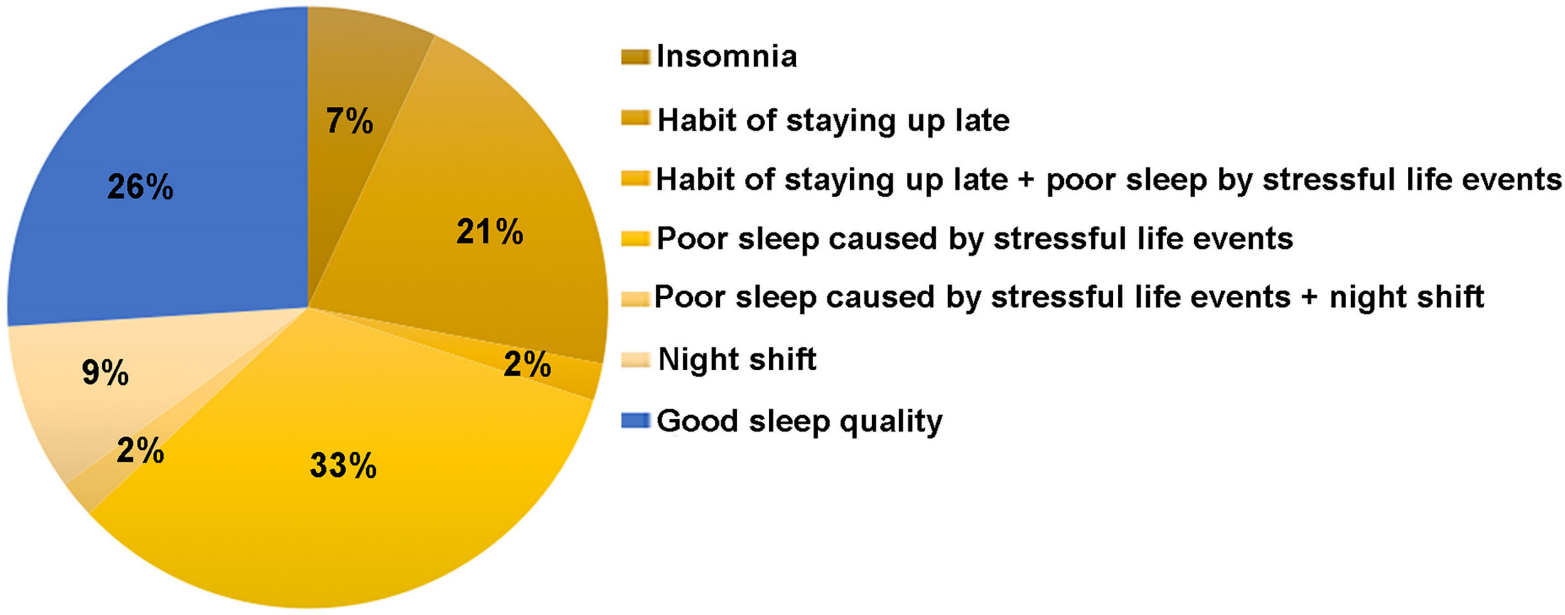
Distribution of sleep status among participants (n = 43).

Sleep problems were reported by most participants, including insomnia, poor sleep caused by stressful life events, the habit of staying up late (usually going to sleep after midnight) and night shift as shown in **Figure 2**. The most common sleep disorder mentioned by participants was poor sleep (including irregular sleep, lack of sleep or decreased sleep quality) caused by stressful life events, such as newborn baby caring (especially for chronically ill child). One participant (*Participant 2*) had secondary amenorrhoea at the age of 33, and was given a diagnosis of POI one year later. She stated,

“My child was born prematurely, and then I always didn’t have enough sleep. I sat up all night with my baby because he kept crying. I think I slept for less than 2 hours a day for almost half a year.”

Another woman with POI (*Participant 37*) experienced menstrual irregularities at the age of 33 and she had the diagnosis of POI at 38 years old. She shared,

“I worked as a migrant worker. Every day I returned from work at 9 to 10 p.m. I Usually went to bed at 12 a.m. If I chatted with my friends, I might not be able to go to bed until 1 to 2 a.m., that kind of schedule had lasted for almost 10 years.”

One participant (*Participant 23*) had a one-year sleep deprivation because of the different sleep habit of her newly married husband, which had caused much sleeping issue to her. She stated,

“My husband’s lifestyle was very different from mine. For example, he stayed up late till 1 or 2 a.m., and his sleep habit interrupted my sleep. The light of his phone and the noise of his walking around the room woke me up regularly. Once I was woken up, I could not fall asleep easily. Then that was very annoying.”

This woman was diagnosed with POI when she was 28 years old, and her menstrual irregularities had lasted for 10 months prior to POI diagnosis.

**Figure 3:**
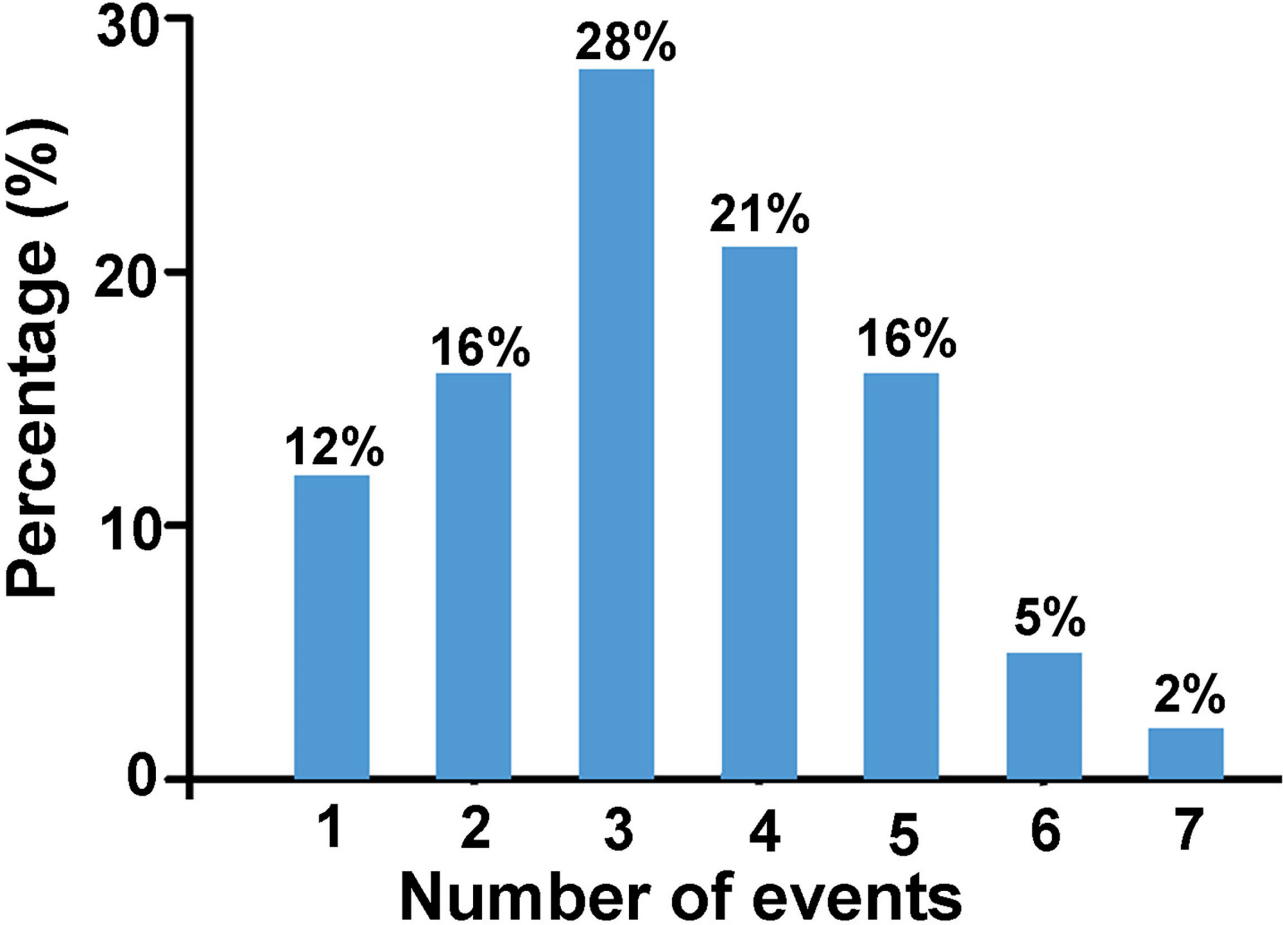
The percentage of women with POI who have experienced certain number of events listed above (n = 43).

Based on the assumption that events are cumulative, that is the more events occur, the greater is the risk (9), we derived the number of events each participant experienced as shown in **Figure 3**. Results showed that 88% of the women with POI experienced two or more stressful life events prior to POI diagnosis.

### Participants’ General Understanding and Concerns About POI

The theme of general information about POI among women with POI mainly focused on “what do you know the effects of POI on your health and how to know?” and “what issues concern you the most after diagnosis”. Most participants said that they did not hear of this disease before diagnosis, while only a few women with POI stated that they knew it was a state of premature menopause through internet or doctors. However, when we asked “Could you know the effects of POI on your health?”, most of them told us that it caused menopause and infertility, but almost no one knew the long-term effects of POI on their health. Participants shared the most concerned thing was fertility in women who had bearing requirement, while for women who had children, the recovery of normal and regular menstruation was the most concern. Of course, how to treatment and whether the ovarian function could be restored were frequently mentioned.

Some with higher education level were eager for the causation of POI. When asked what they thought about the risk factors for POI, a number of responses included, “Could it be my long-term work pressure? Is it caused by staying up late for a long time? Is it because of my long-term depression mood?”

Common misconceptions expressed by the participants included either the use of HRT therapy or herbal medications to treat POI. When mentioned the HRT therapy for POI, most participants were scared that hormone therapy (HT) would be harmful to the body and cause tumor, instead, they and their family believed that Chinese herbal medicine or dietary supplement, such as edible birds nest, piloseantler, or SheueHa (ovaries of Chinese Forest Frog) could treat POI. One of the most common being the sentiment expressed below,

“A friend of mine recommended an old herbalist doctor to me. I have been taking the formula he prescribed for one month. Because it takes a long time for Chinese medicine works, I will stick to the treatment for a period of time.”

## Discussion

Despite the advances in our understanding of POI, about 75-90% of the women with POI remain idiopathic. Based on our observation in gynaecology practice, many women with POI seemed to have a lot of stressful life events. However, to our knowledge, no research has investigated the relationship between stressful/adverse life events and POI. Our qualitative method study was designed to address this issue. Herein, our findings demonstrate that women with idiopathic POI experienced long-term exposure to stressful life events related to workplace stress, family conflicts and sleep disturbance. Furthermore, the participants provided their general understanding and concerns about treatment of POI.

With rapid economic development in China in the past 20 years, immense sociocultural changes have taken place, especially in Shanghai, an international metropolis. Although the income is on the rise, changes in social environment and living condition, such as the increases of cost of living, education and housing, have generated a lot of stress (20). Nowadays, Chinese women are playing more and more important role in employment and financial success than what they did in the past, therefore, it is not a surprise for them to bear more and more social stress (21).

It is known that socio-economic factors can affect age at natural menopause. In particular, early onset of menopause is associated with lower socio-economic status (SES). Factors such as aspects of the physical environment (e.g. crowded living conditions), markers of SES (e.g. lower educational attainment), family dynamics (e.g. early parental divorce) and psychological wellbeing (e.g. feeling overwhelmed or out of control) have all be found to be associated with earlier onset of menopause (22, 23). However, this is the first study to report an association between social environmental factors and the risk of POI.

In our study, “workplace stress” was identified as the most frequent adverse event in women with POI, in which 93% of them were professional/working women and 69% of them were well-educated. Consistent with our finding, Lim YM et al. reported that women in employment were more vulnerable to POI and early menopause compared to those unemployed in a population of Korea (24). In addition, previous studies have also found that long shift at work and stressful workloads were related to menstrual disturbances (25). However, the exact relationship between POI occurrence and progression and workplace stress needs more quantitative study to verify.

Unsurprisingly, high frequency of family-related stressful life events was second to workplace stress. These family related stressful life events include interpersonal conflict among family members, sickness or death of family members, financial constraint and crowded housing issues. Previous studies showed that family stress was detrimental to woman’s mental and physical health, poor relationship quality, relationship dissolution, and death of a loved one are associated with poor psychological and physical health outcomes (26, 27). In China, women are still commonly expected to the primary responsibility of household chores and to care for the offspring and older family members, even if they are in full-time employment. Consequently, working women bear the double burden from their jobs and families, which increases their risk of illness (28). Consequently, as working women, they have the double burden of both work and home, which increased risk of illness among women. Therefore, we will use prospective studies on working women to explore the changes in women’s ovarian function under different workplace stress and life events in the future research.

Lifestyles such as smoking and alcohol are well recognized as risk factors for earlier onset of menopause (29). However, there were no smokers in our participants and only one participant drank occasionally in social activity. Surprisingly, our study showed sleep problems including habit of staying up late, sleep interruption and insomnia were very common in these women with POI. Poor sleep caused by stressful life event/events was related to quality and rhythms of sleep. Previous studies found that short sleep duration and disruption of circadian rhythms were positively associated with menstrual cycle irregularity among women (30). However, it was unknown whether poor sleep quality was associated with the progression of POI disease.

Chronic stressor exposure is considered to cause long-term or permanent changes in emotional, physiological, and behavioral responses that influence susceptibility to disease. A prospective study in patients with multiple sclerosis suggested that high-density life events that occurred during previous year predicted unhealthy outcomes (31). Cumulative stress was related with higher risk of Takotsubo cardiomyopathy, cardiovascular disease and mental health (32). A recent review published in *Science* indicated that adverse social experiences elicited biological responses and influenced health and aging across the life span in social mammals, including human species. Social adversity is one of the strongest predictors of morbidity and mortality risk in humans (33). Our study showed that most women with POI experienced two or more adverse life events prior to POI diagnosis, indicating that cumulative negative effects of environment stressors may play a role in the development of POI.

Participants’ general knowledge and perceptions about POI risk (e.g., etiology, diagnosis, treatment) were another central theme in this study. Unfortunately, most participants had a misunderstanding of POI and were lack of awareness about this condition. Researchers reported that half of women with POI knew the long-time risks of POI in Australia, especially regarding fractures, and most of these women understood benefits of hormone therapy (HT) (34, 35). However, few of women with POI in our study knew the benefit of HT and few took HT before interview. Without prompt and adequate HT, they might develop severe symptoms and long-term health consequences of estradiol deficiency (36–38). Thus, among Chinese women, more investigation should be taken to conduct a survey of disease understanding among a large population of women with POI and appropriate health education should be carried out.

At the end of each interview, we suggested each participant to get rid of the negative events exposed again and build-up healthy lifestyle, regular physical exercise was strongly suggested. Secondly, we recommend hormone therapy promptly and regularly guided by doctors. In order to make information accessible to women with POI, we opened an account in Internet and published popular science articles helping understanding POI. Besides, free doctor’s talks were held in our hospital regularly and women with POI were encouraged to join the health education communications.

## Limitations

Given the qualitative nature of this study, and the sampling methods used, adverse life events as the potential risk factor of POI should be further confirmed by future research. Additionally, further studies are needed to determine whether women with POI in cities of country with different cultures and economic levels respond differently with adverse life events. Thirdly, we did not do the genetic analysis for women with POI except the examination of chromosome and/*FMR1* premutation. Owing to the development of approaches of genome-wide association studies and next-generation sequencing technologies, novel genes might be identified to provide a great opportunity to demonstrate the missing heritability of idiopathic POF, and the identification of specific gene defects will help direct potential targets for future treatment.

## Conclusions

Despite these limitations, our research revealed that cumulative stressful life events might shape the risk of POI and trigger the disease progression. We suggest pre-emptive identification of the at-risk population, especially young Chinese women under increasing pressure and challenges. Tailored interventions aimed to reduce the impact of both work and family stresses among young women are needed to improve their well-being and possibly prevent POI, which may be more cost effective than treating women with POI. In addition, eliminating stress from adverse life events and improving sleep quality will also help to improve the quality of life of women with POI. Besides, POI health education and the benefits of physiologic HRT would help in the management of POI among Chinese women.

## Data Availability Statement

The original contributions presented in the study are included in the article/**Supplementary Material**. Further inquiries can be directed to the corresponding authors.

## Ethics Statement

The studies involving human participants were reviewed and approved by International Peace Maternity and Child Health Hospital (IPMCH) Ethic Committee. The patients/participants provided their written informed consent to participate in this study. Written informed consent was obtained from the individual(s) for the publication of any potentially identifiable images or data included in this article.

## Author Contributions

DL and BL conceived the study, JS, YF, YG, HP and CZ contributed to the study design. DL conducted the interview. JS, YF, YG, HP, TG, GM, YH, LW, BNL participated in audio transcription and data collection, and JS, YF, YG performed the data analysis. DL, JS, YF, YG, QWZ, QW and QZ contributed to blood sample collection. DL, JS, YF drafted the first manuscript and all the authors contributed to subsequent revisions. All authors contributed to the article and approved the submitted version.

## Funding

This study was funded by National Key Research and Development Program of China (No.2018YFC1004800, 2018YFC1004802), National Natural Science Foundation of China (No. 81971334), Shanghai Municipal Education Commission—Gaofeng Clinical Medicine (No. 20152236).

## Conflict of Interest

The authors declare that the research was conducted in the absence of any commercial or financial relationships that could be construed as a potential conflict of interest.

The reviewer JZ declared a shared affiliation with the authors to the handling editor at the time of review.

## Publisher’s Note

All claims expressed in this article are solely those of the authors and do not necessarily represent those of their affiliated organizations, or those of the publisher, the editors and the reviewers. Any product that may be evaluated in this article, or claim that may be made by its manufacturer, is not guaranteed or endorsed by the publisher.

## Abbreviations

POI/POF, Primary ovarian insufficiency/failure; AMH, anti-Mullerian hormone; FSH, follicle-stimulating hormone; LH, luteinizing hormone; E2, estradiol; AFC, Antral follicle count.
