# Supplementary material for: Chronic and Cumulative Adverse Life Events in Women with Primary Ovarian Insufficiency: An Exploratory Qualitative Study
Source: Front Endocrinol (Lausanne). 2022 Jun 23;13:856044. doi: 10.3389/fendo.2022.856044 (PMC9259945; doi:10.3389/fendo.2022.856044)
Supplement: Supplementary file 1 [file DataSheet_1.docx]

**Topic Guide**

**Opening section**

- Welcome, introduction and icebreaker question
- Check consent for audio recording
- Reminding the right to withdraw

**Main section**

**Broad topic 1: Family relationship**

The main purpose is to explore the link between adverse family relationship and the onset of POI, examples of used questions were:

What is your marital status?

To probe:

Do you think your relationship with the other half was good before the diagnosis of the POI disease? Did you have a good relationship with other family members before the diagnosis of the POI disease?

To probe:

In your view, what have been the reasons for this unsatisfied relationship, and how long has it been like this?

**Broad topic 2: Occupation and income**

The main purpose is to explore the link between stress from occupation and income, and the onset of POI, examples of used questions were:

Were you satisfied with your work environment, security and wages before the diagnosis of the POI disease?

To probe:

Did you like your occupation? Did you think that you were under great work stress? How long has it been like this?

Did you need to work long days? Did you have enough time to rest?

How was your relationship with senior and junior colleagues at work?

Could your income meet essential living costs?

**Broad topic 3: Severe social life events and perceived exposure to environmental pollutants**

The main purpose is to explore the link between severe social life events and perceived exposure to environmental pollutants, and the onset of POI, examples of used questions were:

Would you describe your social relationship with friends, neighbors and other people harmonious before the diagnosis of the POI disease?

Did you have any contact with known chemical agents (e.g., pesticides, industrial pollutants, consumer products, and medications) or physical agents (e.g., radiation from medical and other environmental sources) prior to the diagnosis of the disease?

To probe:

Had you involved in or experienced any significant events like serious diseases, legal disputes/cases or death prior to the diagnosis of the disease? When did it happen? How has that affected you?

**Broad topic 4: Life style**

The main purpose is to explore the link between life style factors and the onset of POI, examples of used questions and probes were:

How would you describe your eating habit prior to the diagnosis of the disease?

How often did you consume alcoholic drinks and how much did you consume each time?

In a typical week, how many times did you engage in moderate (such as brisk-walking) to vigorous (such as running) physical activities? How long did each exercise session last usually?

**Broad topic 5: Sleep**

The main purpose is to explore the link between sleep condition and the onset of POI, examples of used questions and probes were:

Did you have a sleep problem prior to the diagnosis of the disease?

Do you think your sleep problems were linked to the negative events mentioned above?

**Broad topic 6: Perceptions of POI**

The main purpose is to understand patients’ knowledge about the disease POI, examples of used questions and probes were:

Have you ever heard of POI? What do you know about the effects of POI on your health? What issues have concerned you the most since diagnosis? What’s your opinion on HT (Hormone therapy)? Do you adhere to medication? (If no, clarify why.)

**Ending section**

- Summary of main points discussed
- Member checking
